# Ultrasonic extraction of anthocyanins from *Lycium ruthenicum* Murr. and its antioxidant activity

**DOI:** 10.1002/fsn3.1542

**Published:** 2020-04-27

**Authors:** Zenggen Liu, Xiaohong Tang, Chuang Liu, Banmacailang Dong, Yun Shao, Baolong Liu, Huilan Yue

**Affiliations:** ^1^ Key Laboratory of Tibetan Medicine Research Northwest Institute of Plateau Biology Chinese Academy of Sciences Xining China; ^2^ Qinghai Provincial Key Laboratory of Tibetan Medicine Research Xining China; ^3^ Qinghai Ruihu Biological Resources Development Co., Ltd Xining China; ^4^ Key Laboratory of Adaptation and Evolution of Plateau Biota Northwest Institute of Plateau Biology Chinese Academy of Sciences Xining China

**Keywords:** anthocyanin, antioxidant activity, *Lycium ruthenicum* Murr., response surface methodology, ultrasonic extraction

## Abstract

The ultrasonic extraction (UE) technology, possessed the advantages of effective, energy‐saving, and environmental‐friendly, was applied to extract the anthocyanin from *Lycium ruthenicum* (LR). The extraction parameters of UE were optimized by response surface methodology (RSM) with Box–Behnken design (BBD). Anthocyanin composition in LR fruits grown in China was systematically identified and quantified by HPLC‐ESI‐MS. The result showed that PRG was the major anthocyanin, and delphinidin, petunidin, and malvidin were the major anthocyanidins in LR fruits. There was the same anthocyanin composition of LR and great variation in anthocyanins content of LR from different areas in China. However, there was no significant difference between wild and cultivated LR in the same region. A clear separation of LR according to geographical origins was revealed by hierarchical cluster analysis (HCA) and principal component analysis (PCA), and the discrimination model for the anthocyanin concentrations were developed using these two analysis methods. Furthermore, on‐line HPLC‐DPPH assay and scavenging activity of three kinds of radicals (DPPH·, ·OH, and $O_{2}^{-\cdot }$) in vitro were well applied to evaluate the antioxidant activity of the LR anthocyanin extract (LRAE). And its results indicated the LRAE could be a credible antioxidant agent for applications in cosmetics, food, and medicine.

## Graphical abstract

Anthocyanin composition in *Lycium ruthenicum* fruits was systematically identified and quantified by HPLC‐ESI‐MS. The antioxidant activity of the LR anthocyanin extract was evaluated by on‐line HPLC‐DPPH assay and scavenging activity of three kinds of radicals in vitro.

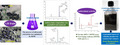

## 1 INTRODUCTION

*Lycium ruthenicum* (LR) is a medicinal and edible fruit tree, and usually grows in the habitat of saline‐alkali, arid, and desert region (Liu et al., 2012; Wang, Li, et al., 2018). Since LR has high content of functional constituents such as anthocyanins, flavonoids, polysaccharides, pectin, and oils (Liu et al., 2019; Peng, Liu, Shi, & Li, 2014; Zheng et al., 2011), the whole plant has been used as a traditional medicine to treat abnormal menstruation, menopause, eye disease, and hypertension, as documented in the classical “The Four Medical Tantras” “Compendium of Materia Medica” (Liu et al., 2012; Wang, Li, et al., 2018). Of these components, anthocyanins have a wide range of physiological and biological activities (Chen et al., 2019; Wang, Li, et al., 2018; Zhang et al., 2019). Therefore, the LR anthocyanin composition and content should be systematically investigated and analyzed, which is important for further commercial utilization and the medical development of LR. Some papers reported the anthocyanin composition of LR (Nzeuwa et al., 2017), and malvidin‐3,5‐di‐O‐glucoside (Zheng et al., 2011) or cyanidin‐3‐glucoside (Wang, Yan, et al., 2018) was applied as the standard compound to semi‐quantify the anthocyanins. However, few researches related to systematical determination and quantitative analysis of the content of anthocyanin and anthocyanidin in LR from different regions were reported. And there was no report for comparative assessment of anthocyanin content in wild and cultivated LR fruits.

In recent years, it was becoming increasingly interested and prevalent in the study field of extraction of anthocyanins from vegetables and fruit (Demirdöven, Özdoğan, & Erdoğan‐Tokatlı, 2015). Some anthocyanin extraction methods are inefficient, high energy consumption, and time‐consuming, and anthocyanins are easily degradable at higher extraction temperatures. Thus, developing new extraction methods with higher efficiency and yields in anthocyanin extraction is a key focus and of great importance in industrial applications of anthocyanin. Based on our previous work, ultrasonic extraction (UE) was considered as an efficient and green technique for extracting anthocyanins from LR. The main UE processing parameters were optimized for extracting anthocyanin by RSM with BBD. Moreover, a highly sensitive on‐line HPLC‐DPPH assay was performed for study on antioxidant activity of LRAE, and LRAE radicals scavenging activity (DPPH·, ·OH, and $O_{2}^{-\cdot }$) in vitro were also investigated. Thus, the optimal UE conditions of LRAE were obtained, and main characteristic constituents and comparative evaluation of anthocyanin content in wild and cultivated LR were clearly clarified. And it was also demonstrated that LRAE presented strong antioxidant capacity, which could be suitable for product development in the field of cosmetics, food, and medicines.

## 2 MATERIALS AND METHODS

### 2.1 Materials and chemicals

The ripe *L. ruthenicum* fruits were collected from Northwest China, which represented the majority of natural populations in their genuine producing areas. Their originations and distributions were shown in Figure S1. Based on on‐line HPLC‐DPPH bioactivity‐guided assay, the high purity of the anthocyanin compound, petunidin‐3‐*O*‐rutinoside (*trans‐p‐*coumaroyl)‐5‐*O*‐glucoside (PRG), was successfully prepared from the LR fruits through extraction and purification by chromatography of macroporous resin, silica gel, and reversed‐phase C18 column. The spectral data of PRG compound were consistent with the previous reports (Jin, Liu, Guo, et al., 2015; Jin, Liu, Yang, et al., 2015; Tang et al., 2017). BHT, ascorbic acid, delphinidin (CAS: 528‐53‐0), petunidin (CAS: 1429‐30‐7), and malvidin (CAS: 643‐84‐5) were purchased from Macklin. HPLC grade solvents including acetonitrile and methanol were purchased from Xinlanjing chemical industry Co., Ltd. All other chemical reagents were of analytical grade and purchased from Sigma‐Aldrich Co.

### 2.2 The extraction methods of anthocyanin

The process of ultrasonic extraction (UE) was as follows. A sample of 5.0 g of powdered LR fruits was conducted in an ultrasonic extractor (JP Ultrasonic 180ST, Skymen Cleaning Equipment Co., Ltd.) using 100 ml ethanol concentration of 90% for 30 min. The working power and temperature were fixed at 300 W and 40°C, respectively. Microwave‐assisted extraction (MAE) was prevalent for extraction of anthocyanins in recent years. 5.0 g of ground LR powders was mixed with 100 ml 90% ethanol in a glass flask and then placed in a MG08S‐2B microwave extraction apparatus. The microwave power and extraction time were fixed at 450 W and 25 min, respectively. Soaking extraction (SE) was a traditional method for extraction of natural products at room temperature, especially for extraction of the heat‐labile compounds. In this study, 5.0 g of powdered LR fruits were mixed with 100 ml 90% ethanol in a glass flask and then dissolved by using a magnetic stirrer for 10 min. During the process of SE, the extracting solution was stirred 5 min for every 1 hr, and the total extraction time was 4 hr. After the extraction with the three methods, the extracting solutions were centrifuged at 5,000 *g* for 20 min to collect the supernatant, and the insoluble residues were treated again for two times as mentioned three extraction methods, respectively. The supernatant was incorporated and concentrated on a rotary evaporator (EYELA) at 50°C. And the extracts obtained were redissolved in ethanol and then filtered through a 0.45 μm reinforced nylon membrane filter for HPLC analysis and the study of antioxidant activity.

Three extraction times were carried out in all the extraction methods, and the successive anthocyanin extracts were incorporated and analyzed. The anthocyanin extract yield (%) was gravimetrically calculated by the following equation: anthocyanin extracts yield (%) = M_anthocyanin extracts_/M_dried LR fruits_ × 100%. The content of anthocyanin (PRG, delphinidin, petunidin, and malvidin) was quantitatively determined and analyzed by HPLC (Figure S2). The values of anthocyanin extracts yield (%) and anthocyanin content were the average of triplicate in each trial.

### 2.3 Infrared spectroscopy of LR fruits

*Lycium ruthenicum* fruit samples were mixed with potassium bromide powder which was spectroscopic grade. And then, 1 mm pellets were formed through pretreatment of the mixtures for Fourier transform infrared (FT‐IR) measurement and analysis. A Bruker TENSOR 27 FT‐IR spectrometer in the frequency range of 4,000–400 cm^−1^ was applied to determine and characterize the FT‐IR spectrum of the LR samples.

### 2.4 Optimization of extraction conditions for anthocyanin extract

Based on the previous single‐factor tests, the experimental factors for ultrasonic extraction of LRAE were optimized by RSM with Box–Behnken design (BBD) of four variables and three levels. As shown in Table S1, the extraction process at a four‐variable, three‐level BBD was assessed by using four parameters, extraction power (W, *X*_1_), extractant (90% ethanol)‐material ratio (mL/g, *X*_2_), temperature (°C, *X*_3_), and time (min, *X*_4_). Design‐Expert software (Version 7.0.1.0) was used to analyze the RSM data and determine the best combination of extraction parameters for the production of anthocyanin extract. The model and theory with second‐order polynomial have been explained and developed in our previous research (Liu et al., 2013), and its simplified model is exhibited by the following equation.

$$Y=\alpha_{0}+\sum_{m}\alpha_{m}X_{m}+\sum_{m}\alpha_{\mathrm{mm}}X_{m}^{2}+\sum_{m}\sum_{n}\alpha_{\mathrm{mn}}X_{m}X_{n}$$

where *Y* (%) is the content of PRG in anthocyanin extract, and it is the predicted response; *α*_0_ is a constant; *α_m_* is the first‐order model coefficient; *α_mm_* stands for the squared coefficient for the factor *m*; the linear model coefficient for the interaction between factors *m* and *n* was displayed by *α_mn_*; and two independent variables were showed by *X_m_* and *X_n_* (*m* ≠ *n*).

### 2.5 On‐line HPLC‐DPPH assay

**FIGURE 1:**
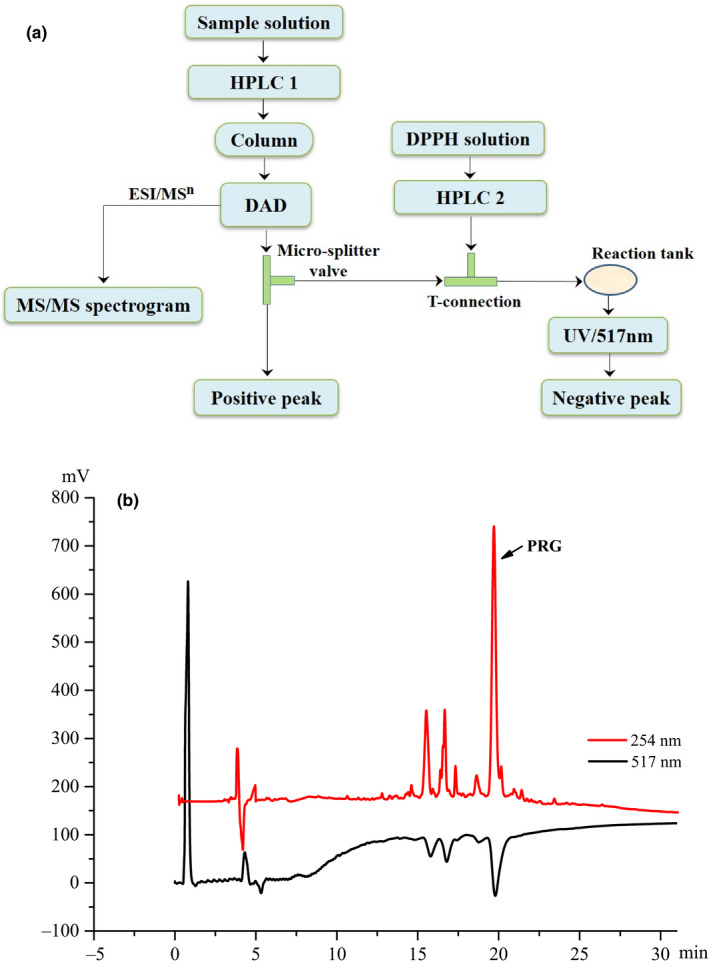
(a) Instrument set‐up of on‐line system; (b) The HPLC profiles (254 nm) and the DPPH radical scavenging profiles (517 nm) of the anthocyanin extract

This assay was performed using the same two preparative Agela P 2010 system with two binary gradient pumps, a diode array detector (DAD) and a visual web detector (VWD), a sample collector and three triple valves, and Agela HPLC software (Agela; Figure 1). The C18 column of Megres (250 × 4.6 mm, 5 μm i.d., Hanbon) was carried out for the separation and analysis of LRAE. The reaction coils were assembled by polyetheretherketone (PEEK) tubing (15.0 m × 0.25 mm i.d.). The antioxidant compounds presented in the LRAE were comprehensively analyzed and identified by a HPLC‐MS (Agilent 1100 Series LC/MSD Trap, Agilent Technologies) fitted with an electrospray ionization (ESI) source.

### 2.6 Determination and evaluation of antioxidant capacity

The scavenging activity of three kinds of radicals (DPPH·, ·OH, and $O_{2}^{-\cdot }$) in vitro was used for determination and evaluation of the antioxidant activity of anthocyanin extract samples, which was described and practiced by the previous researches. The effect of anthocyanin extract on DPPH radical was detected according to the method described by Geng, Chi, Dong, and Hu (2015) and Brandwilliams, Cuvelier, and Berset (1995). Measurement of superoxide anion ($O_{2}^{-\cdot }$) scavenging activity was performed referring to the procedures of Lin and Li (2010). In addition, the assessment and analysis of scavenging activity of hydroxyl radical was usually based on the Fenton‐type reaction (Morales, 2005). All these methods with some modifications were used to determine and evaluate the antioxidant activity, and each test was assayed in triplicates, and then, the experimental data were subjected to an analysis of variance for a completely random design.

## 3 RESULTS AND DISCUSSION

### 3.1 Effects of extraction parameters

The three methods, UE, MAE, and SE, were popularly used for extraction of anthocyanin from LR fruits. However, in this study, UE was the best among these three methods, which indicated the anthocyanin extract yield of 17.92% and the PRG content of 27.66 mg/g DW (Table 1). Thus, in the UE process, it was greatly considered as the most important intention for the selection and combination of potentially effective extraction factors. For the optimization of UE conditions via RSM, all the LR fruit samples were cultivated and collected from GEM, Qinghai. Through the previous analysis of single‐factor experiments, four parameters, extraction power, extractant (90% ethanol)‐material ratio, temperature, and time, were selected as the effective UE factors. And these extraction conditions played a great role in the extraction process for the improvement of extraction efficiency and selectivity of extractant. Thus, the extraction parameters, 300 W, 20 ml/g, 40°C, and 45 min were employed for the central point of the RSM tests, respectively.

**TABLE 1:** Anthocyanin extract yield and PRG content of LR fruits in comparison with different extraction methods

Extraction method	Anthocyanin extract yield (%)	PRG content (mg/g DW)
UE	17.92 ± 0.93	27.66 ± 0.81
MAE	16.85 ± 0.80	26.15 ± 1.28
SE	16.34 ± 1.17	24.78 ± 0.55

Note

The fruit samples were cultivated and collected from GEM, Qinghai.

Abbreviations: MAE, microwave‐assisted extraction; SE, soaking extraction; UE, ultrasonic extraction.

### 3.2 Optimization of ultrasonic extraction parameter via RSM

In this study, 29 different combinations of anthocyanin (PRG) content were tested and showed in Table S1. The response value was correspondingly obtained by each extraction combination of the extraction parameters. ANOVA was applied to test and analyze the regression coefficients of the linear, intercept, two‐factor interactions, and quadratic polynomial of the model. As shown in Table 2, the result indicated that this analysis model was appropriate for prediction and optimization of the UE factors. Additionally, based on ANOVA of experimental data, Pareto chart (*p* = 95%) presented the effects of factors and factor interactions were exhibited in Figure S3. The effects of the variables estimated with the dashed line on the plot were statistically significant (*p* < .05). The green response meant the positive sign was enhanced by the variable, while the blue plot response represented negative sign was reduced by each variable. It was highly significant (*p* < .01) that four linear parameters (*X*_1_, *X*_2_, *X*_3_, and *X*_4_) and all quadratic parameters were presented in the model. Combining with multiple regression analysis, a relationship between the response (PRG content of LR fruit) and variables was obtained and expressed by the following second‐order polynomial equation:

$$\begin{array}{cc} Y & =27.74+0.56X_{1}+0.91X_{2}+0.53X_{3}+1.16X_{4}+0.11X_{1}X_{2}\\ & -0.29X_{1}X_{3}-0.34X_{1}X_{4}-0.36X_{2}X_{3}-0.16X_{2}X_{4}\\ & -0.15X_{3}X_{4}-0.87X_{1}^{2}-0.83X_{2}^{2}-0.61X_{3}^{2}-1.17X_{4}^{2} \end{array}$$

where *Y* (mg/g) is the response value of PRG content, *X*_1_, *X*_2_, *X*_3_, and *X*_4_ are the means of the variables, extraction power, extractant‐material ratio, temperature, and time, respectively.

**TABLE 2:** ANOVA of the quadratic polynomial model for the PRG content via UE

Parameter	Coefficient estimate	Standard error	Sum of squares	*df*	Mean square	*F*‐value	*p* > *F*
Model			48.76	14	3.48	15.24	<.0001
Intercept	27.74	0.21		1			
*X*_1_	0.56	0.14	3.73	1	3.73	16.32	.0012
*X*_2_	0.91	0.14	10.01	1	10.01	43.80	<.0001
*X*_3_	0.53	0.14	3.38	1	3.38	14.80	.0018
*X*_4_	1.16	0.14	16.24	1	16.24	71.06	<.0001
*X*_1_ * X*_2_	0.11	0.24	0.051	1	0.051	0.22	.6451
*X*_1_ * X*_3_	−0.29	0.24	0.35	1	0.35	1.52	.2375
*X*_1_ * X*_4_	−0.34	0.24	0.45	1	0.45	1.96	.1828
*X*_2_ * X*_3_	−0.36	0.24	0.53	1	0.53	2.30	.1516
*X*_2_ * X*_4_	−0.16	0.24	0.11	1	0.11	0.48	.5013
*X*_3_ * X*_4_	−0.15	0.24	0.090	1	0.090	0.39	.5404
$X_{1}^{2}$	−0.87	0.19	4.96	1	4.96	21.68	.0004
$X_{2}^{2}$	−0.83	0.19	4.47	1	4.47	19.57	.0006
$X_{3}^{2}$	−0.61	0.19	2.39	1	2.39	10.44	.0060
$X_{4}^{2}$	−1.17	0.19	8.88	1	8.88	38.88	<.0001
Residual			3.20	14	0.23		
Lack of fit			2.58	10	0.26	1.68	.3255
Pure error			0.61	4	0.15		
*SD*	0.48		*R* ^2^	.9384			
Mean	26.30		Adj *R* ^2^	.8768			
C.V.%	1.82		Pred *R* ^2^	.6950			
PRESS	15.85		Adeq precision	12.339			

Furthermore, the analysis model displayed *F*‐value 15.24 (*p* < .01) and coefficient of determination (*R* ^2^) .9384, while predicted *R* ^2^ .6950 was in reasonable agreement with the adjusted *R* ^2^ .8768. These demonstrated that the model was significant. The “lack of fit *F*‐value” was 1.68, which indicated the Lack of Fit was not significant (*p* > .10) relative to the pure error. And the “lack of fit *F*‐value” with a 32.55% chance could occur due to noise. “Adeq Precision” measured the signal to noise ratio, and it was reported that a ratio >4 is desirable (Liu et al., 2013). The experimental ratio of 12.339 implied an adequate signal. Overall, this model was accurate and perfectly applied to navigate the design thought and space.

Three‐dimensional plot for the influence of parameters combination was showed in the Figure S4. With increase in examined variables such as extraction power and time, the positive effects were detected on the predicted PRG content, and the content can reach maximum at 28.32 mg/g at extraction power (*X*_1_) of 347.82 W, extractant‐material ratio (*X*_2_) of 24.85 ml/g, temperature (*X*_3_) of 41.84°C and time (*X*_4_) of 29.17 min. In the convenience issues (such as operating and recording) to consider, the optimum experimental parameters were selected as follows: *X*_1_, 348 W; *X*_2_, 25 ml/g; *X*_3_, 42°C; *X*_4_, 29 min. The PRG content was 28.54 ± 0.86 mg/g (*n* = 3) with using above conditions combination, and there was no significant (*p* > .05) difference between predicted and measured values. Thus, it can be summarized that the RSM model was perfect and adequate to reflect the expected optimization of UE process for the PRG content of LR fruit.

### 3.3 Fingerprints of LRAE

The HPLC and IR fingerprints of LR anthocyanins in the fruit samples were initially analyzed using HPLC‐DAD at a wavelength of 525 nm and FT‐IR spectrometer at the frequency range of 4,000–400 cm^−1^, respectively (Figure S5). In the study of HPLC fingerprint, the chromatograms exhibited the peaks with broad ranges and good resolution was obtained at these present chromatographic conditions, and the common characteristic peaks were selected as analysis of fingerprint. The anthocyanin fingerprint of LR from the different regions was similar. Moreover, all the chromatograms displayed the same anthocyanin composition patterns of LR from different places, whether the LR fruit samples were wild or cultivated. Two main peaks (I and II) were detected in all of the samples, and they were petunidin‐3‐O‐galactoside‐5‐O‐glucoside (molecular ion *m*/*z* 641, fragmentation pattern *m*/*z* 479/317), and PRG (molecular ion *m*/*z* 933, fragmentation pattern *m*/*z* 771/641/479/317), which indicated that the chemical information of the two compounds was consistent with previously published (Zheng et al., 2011). Our study is the first to publish the anthocyanin profile of the LR from Qinghai, Gansu, Xinjiang, Inner Mongolia, and Ningxia. Some direct evidences were provided in our research and indicated that anthocyanin composition patterns are controlled genetically. It was reported that one allelic gene *LrAN2* was isolated from *L. ruthenicum*, which carried the function regulating anthocyanin biosynthesis and accumulation as the MYB transcription factor. High anthocyanin content in the LR fruit could be attributed to the functional diversity and high expression level of *LrAN2* (Zong et al., 2019a,2019b).

Additionally, the LR anthocyanin extracts (LRAE) were scanned in the range of 4,000–400 cm^−1^ for three or more times. Based on the FT‐IR spectrum, signals in the regions of 3,500–3,200 cm^−1^ were attributed to hydroxyl groups stretching and bending vibrations; the small band at around 2,930 cm^−1^ was due to C‐H stretching vibrations. The bands around 1,736 and 1,640 cm^−1^ suggested the presence of the ester carbonyl groups (C=O) and C‐O groups stretching band (Gnanasambandam & Proctor, 2000), and the absorption of the C‐O group at 1,640 cm^−1^ also showed that it was part of glycosides; the absorption peaks between 1,280 and 920 cm^−1^ indicated that galactose conformation of LR anthocyanin was of the pyranose type (Ye et al., 2011); pyranoses existed in the β‐configuration, which were inferred from the weak absorbance at 895 cm^−1^ (Mathlouthi & Koenig, 1987). These results could show that LRAE existed abundantly sugar conjugates which might be glucoside, rutinoside, and galactoside. The characteristics of FT‐IR spectra were analyzed and reported firstly in LR fruit samples from Qinghai, Gansu, Xinjiang, Inner Mongolia, and Ningxia. Furthermore, the IR fingerprints of LR from the different regions were similar, whether the fruit samples were wild or cultivated. Therefore, LR from other species could be probably distinguished, with the unique HPLC and IR fingerprints of LRAE.

### 3.4 Comparison and analysis of anthocyanins content

Three anthocyanidins (delphinidin, petunidin, and malvidin) were obtained during the LR anthocyanin extracts hydrolysis in boiling water bath, and then, the quantification and comparison of four compounds (PRG, delphinidin, petunidin, and malvidin) were systematically investigated (Table 3 and Table S2). In the previous work, 16 anthocyanins from LR fruits were identified, with petunidin derivatives constituting approximately 97% of the total anthocyanin content (Jin, Liu, Guo, et al., 2015; Jin, Liu, Yang, et al., 2015; Wang, Yan, et al., 2018). It was notable that PRG accounted for almost 80% of the total anthocyanins, which was seldom in the common edible berry to our knowledge (Wu et al., 2006; Zheng et al., 2011). Although the exact reasons have not been clarified yet for the high content of PRG and petunidin in LR fruits, it has been speculated that the synthesis and accumulation of anthocyanins were greatly influenced by genomic difference and unique eco‐geographical environments (high altitude, drought, cold and strong sunshine). In this work, PRG consequently proved to be the predominant anthocyanin in LR fruits (Figure S2). The wild LR samples which come from KeluKehu, Qinghai Province, have the highest PRG content (31.51 ± 1.73 mg/g). And the cultivated samples located at Jinta, Gansu Province, have the lowest PRG content (8.29 ± 0.27 mg/g). PRG content ranged from 9.78 ± 0.59 to 31.51 ± 1.73 mg/g in wild samples, and from 8.29 ± 0.27 to 28.54 ± 0.86 mg/g in cultivated samples. In addition, the wild fruit samples in PRG content had significant differences between Qinghai and other Provinces, and the average PRG content varied in the following order, Qinghai (24.71 mg/g), Xinjiang (16.25 mg/g), Inner Mongolia (15.51 mg/g), Gansu (14.42 mg/g), and Ningxia (13.70 mg/g). The PRG content of cultivated fruit samples showed the similar trend in these different provinces. This indicates that LR fruits have great variation in PRG content among different regions. However, the total average content of PRG was slightly higher in wild fruits (17.42 mg/g) than in cultivated fruits (17.05 mg/g), but this difference was not statistically significant (*p* > .05).

**TABLE 3:** Estimation of PRG content of wild and cultivated LR fruits located at different regions in China^a^

No.	Locality	Code	Lt (N)	Ln (E)	Al (m)	PRG content (mg/g DW)	
						Wild	Cultivated
1	Xiangride, Qinghai	XRD	36.01	97.89	3,070	21.04 ± 1.05	22.93 ± 0.67
2	Nuomuhong, Qinghai	NMH	36.43	96.25	2,775	22.75 ± 0.84	18.22 ± 1.24
3	KeluKehu, Qinghai	KLKH	37.29	96.86	2,814	31.51 ± 1.73	23.46 ± 1.10
4	Geermu, Qinghai	GEM	36.46	94.90	2,773	26.14 ± 0.94	28.54 ± 0.86
5	Dagele, Qinghai	DGL	36.27	95.45	2,780	18.07 ± 0.81	15.23 ± 0.33
6	Urt Moron, Qinghai	WTMR	36.88	93.13	2,883	28.77 ± 0.73	27.90 ± 0.90
7	Hongliugou, Xinjiang	HLG	39.14	89.98	2,010	9.78 ± 0.59	10.02 ± 0.27
8	Ruoqiang, Xinjiang	RQ	39.06	88.13	840	16.87 ± 0.94	28.24 ± 0.48
9	Hetian, Xinjiang	HT	37.12	79.93	1,430	16.52 ± 0.77	17.39 ± 0.55
10	Kashgar, Xinjiang	KS	39.41	76.09	1,262	11.69 ± 0.85	10.05 ± 0.41
11	Alaer, Xinjiang	ALE	40.54	81.30	1,078	18.72 ± 0.66	16.83 ± 0.86
12	Yuli, Xinjiang	YL	41.29	86.25	887	11.76 ± 1.37	9.42 ± 0.39
13	Turpan, Xinjiang	TUP	42.93	89.20	10	22.43 ± 1.08	20.25 ± 1.13
14	Changji, Xinjiang	CJ	44.10	87.47	493	21.97 ± 1.37	22.28 ± 0.75
15	Jinghe, Xinjiang	JH	44.64	82.85	274	16.53 ± 0.75	16.78 ± 0.42
16	Dunhuang, Gansu	DH	40.11	94.63	1,162	13.85 ± 0.36	15.54 ± 0.84
17	Guazhou, Gansu	GZ	40.52	95.85	1,178	15.01 ± 0.96	15.77 ± 0.66
18	Jiayuguan, Gansu	JYG	39.73	98.18	1,770	17.30 ± 1.03	18.04 ± 0.57
19	Jinta, Gansu	JT	40.38	99.72	1,150	10.55 ± 0.78	8.29 ± 0.27
20	Shandan, Gansu	SD	38.76	101.06	1,773	12.20 ± 0.40	10.89 ± 0.35
21	Minqin, Gansu	MQ	38.64	103.07	1,360	17.58 ± 0.95	17.11 ± 1.20
22	EjinaQi, Inner Mongolia	EQ	42.02	101.06	920	13.45 ± 1.07	13.93 ± 0.31
23	Alxa Youqi, Inner Mongolia	ALYQ	39.21	101.65	1,480	17.93 ± 0.67	16.06 ± 0.24
24	Alxa Zuoqi, Inner Mongolia	ALZQ	39.03	105.67	1,419	15.97 ± 0.58	15.35 ± 0.44
25	Bayan Nur, Inner Mongolia	BYN	40.85	107.22	1,037	14.69 ± 1.14	15.54 ± 0.85
26	Qingtongxia, Ningxia	QTX	38.05	105.79	1,215	14.62 ± 0.97	14.04 ± 0.29
27	Pingluo, Ningxia	PL	38.90	106.58	1,100	12.77 ± 0.80	12.21 ± 0.51

Note

The values of PRG contents were means ± *SD* (*n* = 3).

Abbreviations: Al, altitude; Ln, longitude; Lt, latitude.

^a^ The PRG was obtained by ultrasonic extraction.

The three anthocyanidins (delphinidin, petunidin, and malvidin) were hydrolytic products from LR anthocyanin extracts. Total anthocyanidins contents ranged from 2.09 to 12.05 mg/g, with an average of 6.10 mg/g in wild fruit samples, and ranged from 3.30 to 15.68 mg/g, with an average of 6.81 mg/g in cultivated samples. Additionally, the average content of anthocyanidins increased in the following order: Gansu < Xinjiang < Ningxia < Inner Mongolia < Qinghai (wild); Gansu < Ningxia < Inner Mongolia < Xinjiang < Qinghai (cultivated). However, there was no significant difference between wild and cultivated fruit samples. The average contents of delphinidin, petunidin, and malvidin were 0.27, 5.42, and 0.42 mg/g in wild samples, respectively, which were similar in the cultivated samples (0.30, 5.99, and 0.51 mg/g). This indicates that petunidin is the major component of LR fruit anthocyanidins. Both of wild (10.78 ± 0.37 mg/g) and cultivated (13.81 ± 1.15 mg/g) fruit samples from Geermu, Qinghai Province, have the highest content of petunidin. However, the lowest contents of petunidin in wild and cultivated fruits were from Hongliugou, Xinjiang Province (1.74 ± 0.22 mg/g), and Jinta, Gansu Province (2.91 ± 0.06 mg/g), respectively. This study also indicates that there is great variation in anthocyanidin content of LR fruit samples located at different regions in China.

### 3.5 Hierarchical cluster analysis and principal component analysis

**FIGURE 2:**
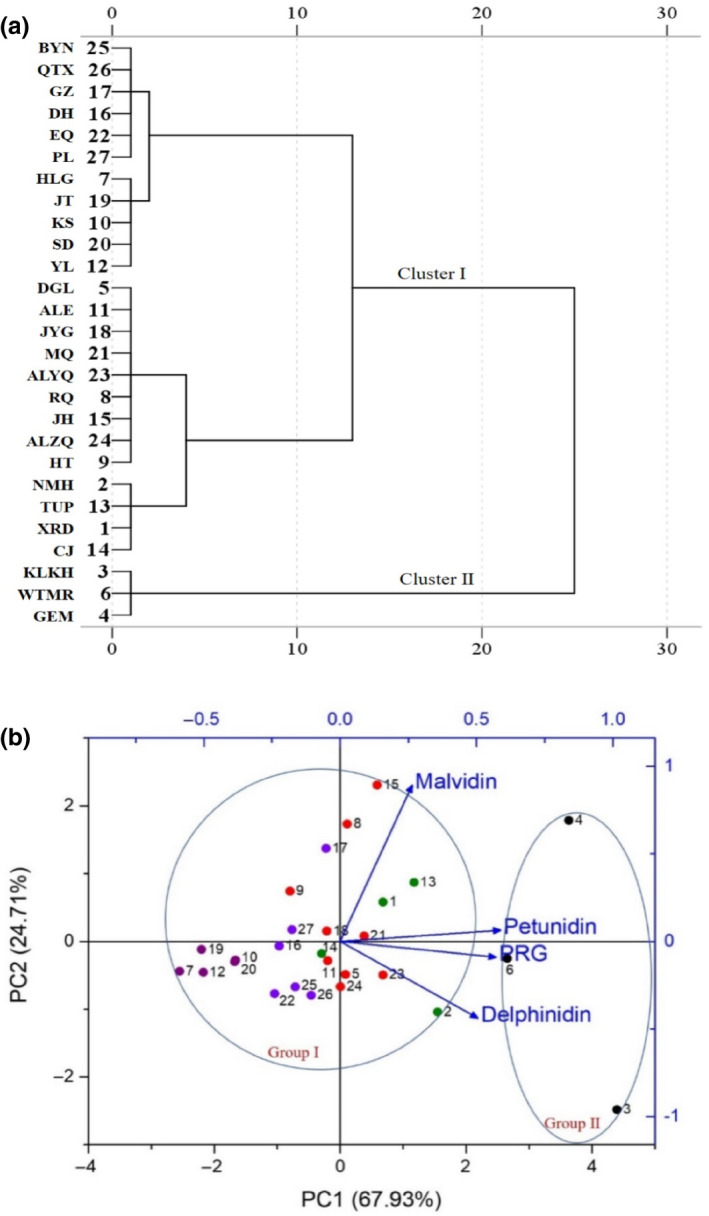
Chemometric analysis of 27 batches of LR. (a) Dendrogram of hierarchical cluster analysis of LR from different regions; (b) Two‐dimensional plot of the anthocyanin content in LR investigated in principal component analysis. The LR fruits samples were wild

Using the quantitative analysis of the four compounds (PRG, delphinidin, petunidin, and malvidin) as input data matrix, a dendrogram was constructed to reveal the relationships among the LR fruit samples as shown in Figure 2a. Through hierarchical cluster analysis (HCA), it was evident that 27 wild LR samples were clearly clustered into two groups as follows: cluster II (**3**, **4**, **6**) and cluster I (the rest of the samples). Cluster I was further divided into two subgroups III (**1**, **2**, **5**, **8**, **9**, **11**, **13–15**, **18**, **21**, **23**, 2**4**) and IV (**7**, **10**, **12**, **16**, **17**, **19**, **20**, **22**, **25–27**). The smallest distance between these two subgroups indicates how smallest and insignificant the differences were.

Principal component analysis (PCA) was firstly carried out using the content of each anthocyanin (PRG, delphinidin, petunidin, and malvidin), which could be helpful to seek further differentiation of the anthocyanin composition in LR according to its geographical origin. The loading plots obtained by PCA are presented in Figure 2b. On the basis of eigenvalues higher than 1, the first two principal components (PCs) accounted for approximately 92.64% of the total variance (PC1 = 67.93%, PC2 = 24.71%). From the loading of the variables presented the scatter points, the samples could be clearly classified into two groups: sample codes **3**, **4,** and **6** consisted of group II, and group I was made up of the rest of the samples. This was consistent with the result of HCA analysis.

### 3.6 Antioxidant activity

To identify the antioxidative compounds in the LRAE, on‐line HPLC coupled with ESI‐MS/MS was used for determination and analysis of the extracted solution. Figure 1a shows the instrument set‐up of on‐line system. A 4:1 splitter was linked up with the DAD, which means one part of HPLC eluent flowed to the mass spectrometer and four parts to the reaction coil. Through this way, MS, UV, and radical scavenging activity data of every peak could be acquired in only one sample injection. It was displayed in Figure 1b that there were several peaks in the chromatograms of LRAE. The main antioxidant peak was identified as PRG. Detailed information of the MS and UV characteristics for this compound was as follows: UV‐vis *λ*_max_ 280 or 530 nm, molecular ion *m*/*z* 933, fragmentation pattern *m*/*z* 771/641/479/317.

**FIGURE 3:**
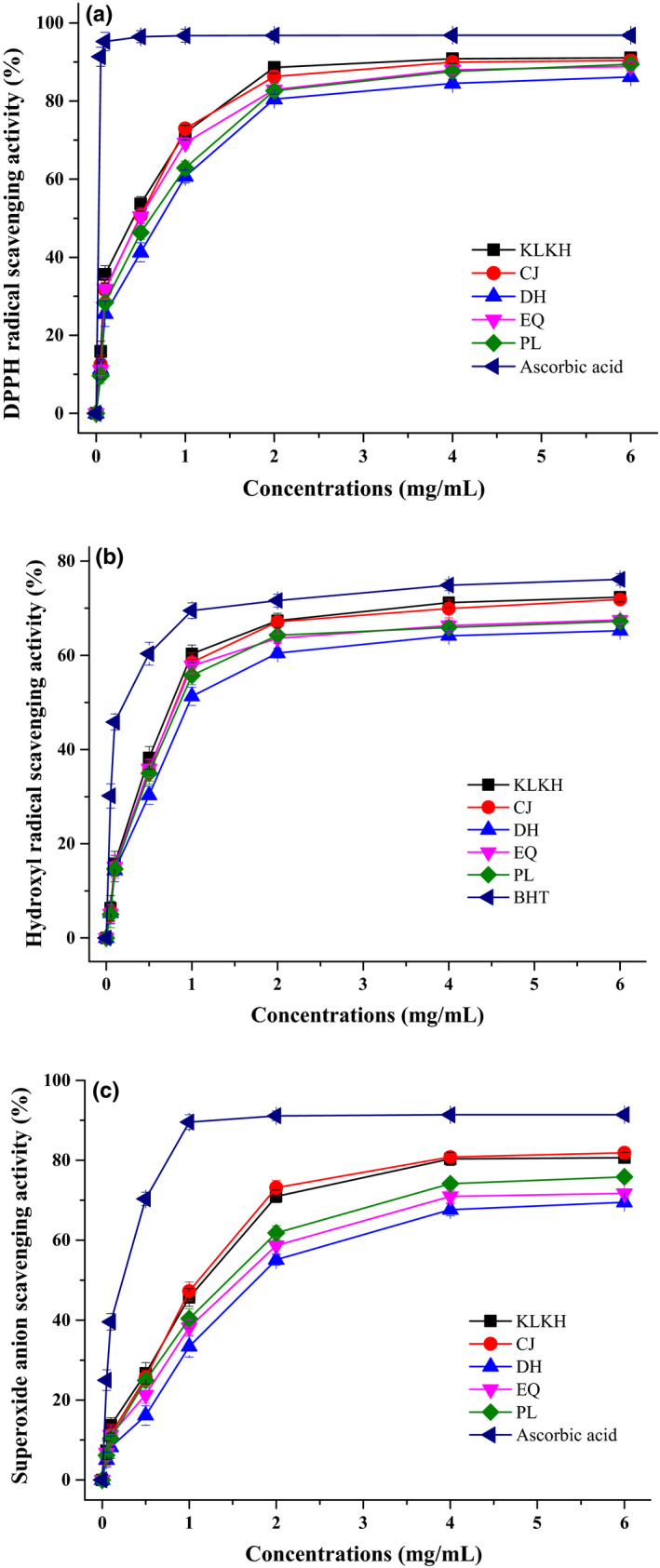
Antioxidant activity of LR anthocyanin extracts from different regions: (a) scavenging activity to DPPH radical; (b) scavenging activity to hydroxyl radical; (c) scavenging activity to superoxide anion; all anthocyanin extracts were obtained from wild LR fruits; values are means ± *SD* (*n* = 3)

The antioxidant activity of LRAE was determined and evaluated by different radicals (DPPH·, OH·) and superoxide anion. These samples were from KLKH, CJ, DH, EQ, and PL, which represented five provinces, Qinghai, Xinjiang, Gansu, Inner Mongolia, and Ningxia, respectively. The DPPH· scavenging activity of anthocyanin extract from wild *L. ruthenicum* is shown in Figure 3a. It indicated the DPPH scavenging activity of 50% at 0.43 mg/ml (KLKH, Qinghai) and 0.77 mg/ml (DH, Gansu), which indicated the minimum and the maximum IC_50_ values, whereas for ascorbic acid was found to be over 80% at 0.05 mg/ml. Moreover, the scavenging activity of DPPH· showed a concentration‐dependent relationship with the anthocyanin extract. The scavenging activity increased at 90.82% (KLKH, Qinghai) while the concentration of anthocyanin extract was 4.00 mg/ml. The dose–response curves for the superoxide radical and hydroxyl radical scavenging activities of anthocyanin extract are described in Figure 3b,c. The percentage of radicals scavenging effects was related well with concentration of LRAE. The LRAE and ascorbic acid for scavenging activity of $O_{2}^{-\cdot }$, were all over 60% at dose of 3.15 mg/ml, and the IC_50_ values were 1.80 (DH, Gansu) and 0.23 mg/ml, respectively. The ·OH scavenging activity of LRAE showed a similar result with $O_{2}^{-\cdot }$. The percentage of ·OH scavenging activity exceeded 60% when both extract and BHT were at 1.98 mg/ml. When the content of LRAE was in the range of 0.05 to 2.00 mg/ml, the scavenging effect increased significantly (*p* < .01). And the IC_50_ values of the anthocyanin extract were 0.78 (KLKH, Qinghai) and 0.96 mg/ml (DH, Gansu), respectively, suggesting that the extracts were good candidates for hydroxyl radical scavenging.

Furthermore, by comparison with the antioxidant activity obtained by wild and cultivated samples (Figure S6), it could be concluded that the cultivated sample from KLKH obtained the highest antioxidant activity in both DPPH· (73.29%) and ·OH assay (62.44%), while DH obtained the lowest antioxidant activity (56.92% and 49.38%, respectively). Significant differences have been detected between other samples and DH samples (*p* < .05). The consequences of $O_{2}^{-\cdot }$ were similar, the samples from KLKH and CJ possessed the high antioxidant activity, and followed by PL, EQ, and DH. Interestingly, there were no significant differences between wild and cultivated samples in all antioxidant capacities (DPPH·, ·OH, and $O_{2}^{-\cdot }$), whether these samples were from KLKH, CJ, DH, EQ, or PL.

## 4 CONCLUSION

In this study, ultrasonic extraction was considered as an efficient, energy‐saving, and environmental‐friendly technique for extraction of anthocyanins from LR. And the extraction process was optimized by RSM with BBD. In LR fruits, PRG was the major anthocyanin, while delphinidin, petunidin, and malvidin were the major anthocyanidins. Anthocyanin composition patterns were found to be the same in all LR fruit samples. There was great variation in anthocyanins content of LR from different areas in China. However, there was no significant difference between wild and cultivated LR fruits in the same region. The results also demonstrated that the anthocyanin composition could be used as an indicator for the determination of LR geographical origin. The differentiations of LR were monitored and enabled by the data of individual anthocyanin contents (PRG, delphinidin, petunidin, and malvidin) together with multivariate statistical analysis techniques. HCA and PCA presented a clear separation of LR according to geographical origins. In addition, the potential antioxidant activity of anthocyanin extract from LR was evaluated by an on‐line HPLC assay and the in vitro test of the free radicals (DPPH·, ·OH, and $O_{2}^{-\cdot }$) scavenging activities. And it was showed that the anthocyanin extract possessed strong capacity of scavenging these radicals. Therefore, LR anthocyanin extracts have the potential to be explored as health‐promoting antioxidant agent applied in the field of food, cosmetics, or pharmaceuticals. However, it still needs to be investigated further with establishment of quality standards and safety evaluation for LR anthocyanin extracts.

## CONFLICT OF INTEREST

In the process of research, the authors have no competition and no conflicts of interest.

## ETHICAL APPROVAL

The work does not involve any human or animal testing.

## INFORMED CONSENT

This study does not require informed consent because it does not use humans as research subjects.

## Supporting information

Figure S1

Figure S2

Figure S3

Figure S4

Figure S5

Figure S6

Table S1

Table S2
