# Supplementary material for: Ultrasonic extraction of anthocyanins from Lycium ruthenicum Murr. and its antioxidant activity
Source: Food Sci Nutr. 2020 Apr 27;8(6):2642–51. doi: 10.1002/fsn3.1542 (PMC7300067; doi:10.1002/fsn3.1542)
Supplement: Supplementary file 2 — Figure S2 [file FSN3-8-2642-s002.docx]

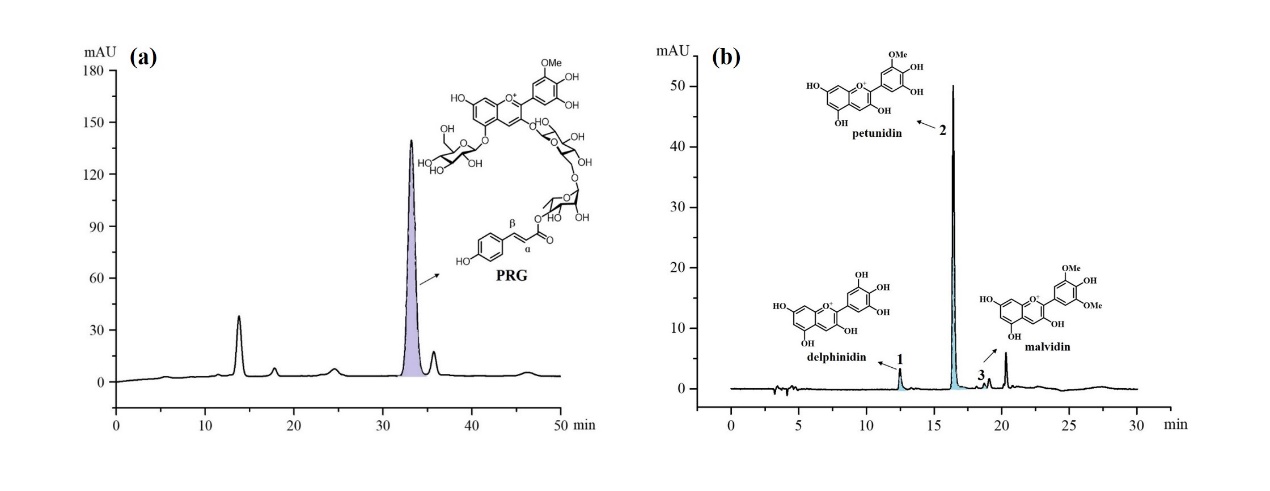


**Figure S2** High performance liquid chromatograms of PRG and anthocyanidins (delphinidin, petunidin and malvidin) extracted from the cultivated fruit of *L. ruthenicum* (GEM, Qinghai). The four compounds were analyzed by SPD-M20A HPLC system (Shimadzu, Japan), equipped with a LC-20AD HPLC pump, a SIL-20AC autosampler, a CTO-20AC thermostated column compartment and a SPD-20AV photodiode array detector. The analytical column was C18 column of Megres (250×4.6 mm, 5 μm i.d., Hanbon, China). An aliquot of 10 μL solution was injected. Chromatograms were obtained at 525 nm, and photodiode array spectra was recorded from 190 to 800 nm. Gradient programs were applied for PRG analysis. The eluents were A (2% of formic acid in water) and B (2% of formic acid in acetonitrile). The applied elution program for separation of PRG was: 0-10 min, linear gradient from 7% to 13% B; 10-30 min, linear gradient from 13% to 15% B; 30-50 min, 15% B isocratic. The flow rate was 1.0 mL/min, and temperature 35 ℃. Additionally, The gradient conditions for separation of anthocyanidins was as follows: 0-2 min, 8-12% B; 2-5 min, 12-18% B; 5-10 min, 18-20% B; 10-12 min, 20-25% B; 12-15 min, 25-30% B; 15-18 min, 30-45% B; 18-20 min, 45-80% B; 20-22 min, 80-8% B; 22-30 min, 8% B. This flow rate was 0.8 mL/min, and temperature 35 ℃. The PRG and anthocyanidins content were calculated by comparison with the values obtained from a standard curve, and expressed in mg/g dry weight (DW).
