# Supplementary material for: Ultrasonic extraction of anthocyanins from Lycium ruthenicum Murr. and its antioxidant activity
Source: Food Sci Nutr. 2020 Apr 27;8(6):2642–51. doi: 10.1002/fsn3.1542 (PMC7300067; doi:10.1002/fsn3.1542)
Supplement: Supplementary file 3 — Figure S3 [file FSN3-8-2642-s003.docx]

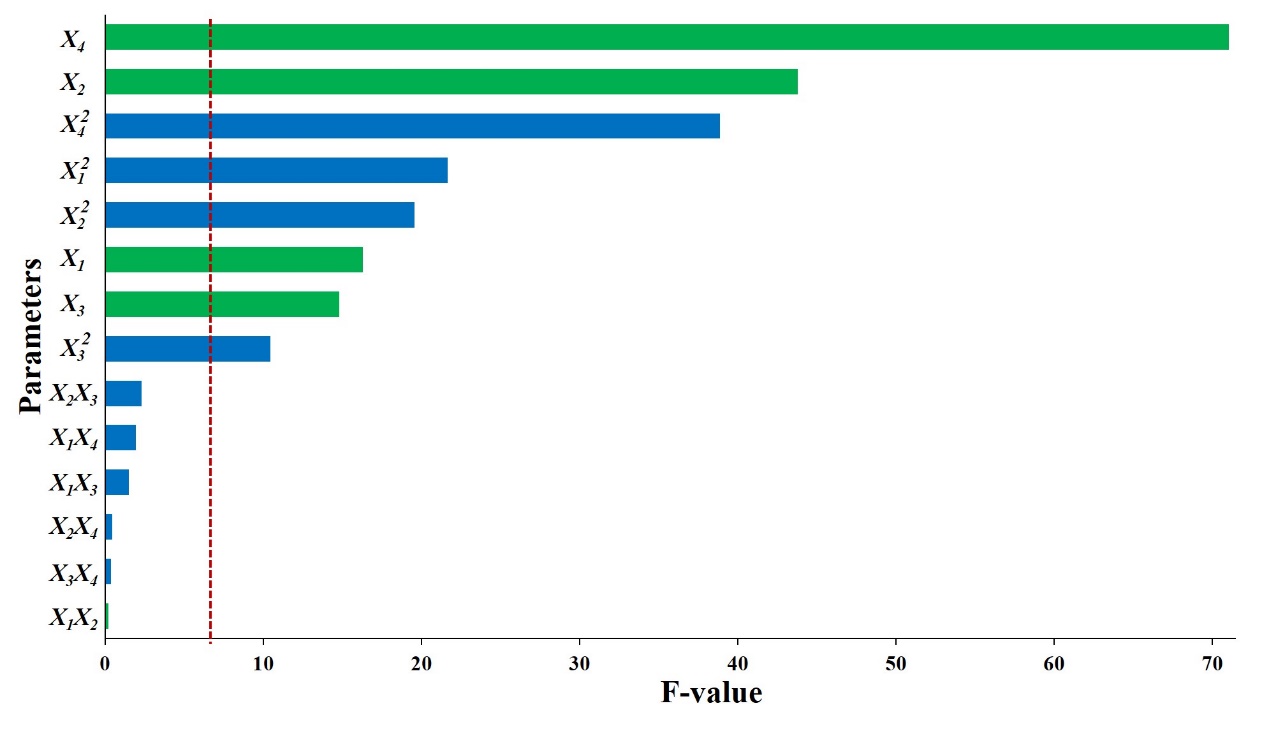


**Figure S3** Pareto chart for the evaluated effects of extraction factors and factor interactions on PRG content.
