# Supplementary material for: Ultrasonic extraction of anthocyanins from Lycium ruthenicum Murr. and its antioxidant activity
Source: Food Sci Nutr. 2020 Apr 27;8(6):2642–51. doi: 10.1002/fsn3.1542 (PMC7300067; doi:10.1002/fsn3.1542)
Supplement: Supplementary file 4 — Figure S4 [file FSN3-8-2642-s004.docx]

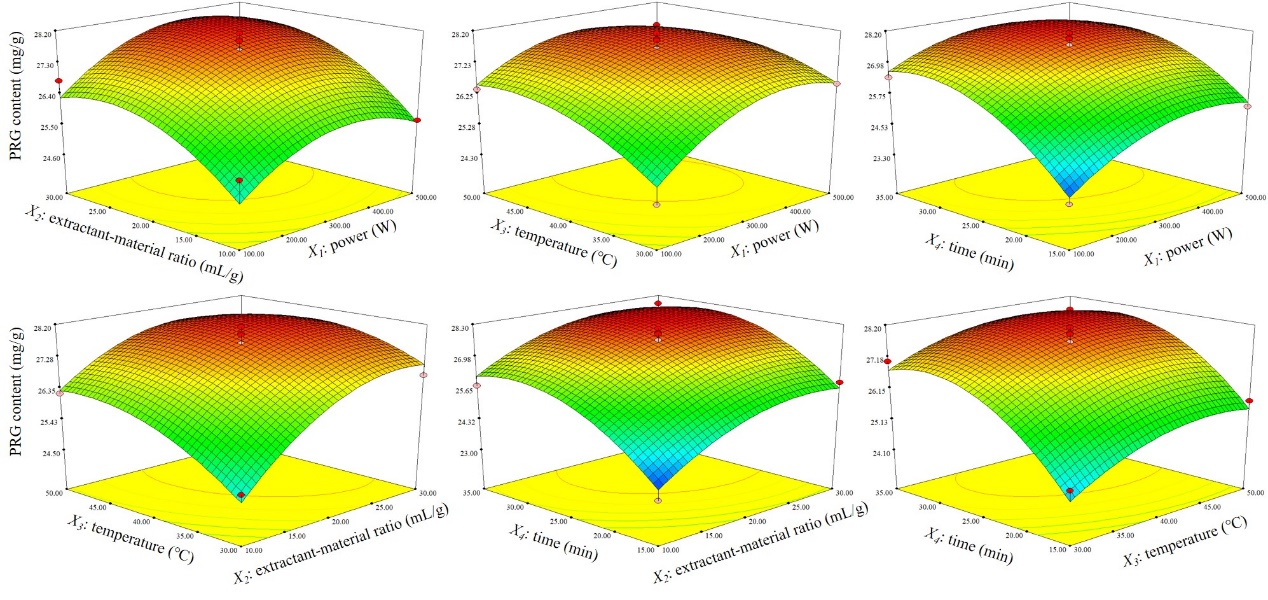


**Figure S4** Response surface plots of the PRG content via UE affected by extraction power (*X_1_*), extractant-material ratio (*X_2_*), temperature (*X_3_*), and time (*X_4_*).
