# Supplementary figures and images for: Ultrasonic extraction of anthocyanins from Lycium ruthenicum Murr. and its antioxidant activity
Source: Food Sci Nutr. 2020 Apr 27;8(6):2642–51. doi: 10.1002/fsn3.1542 (PMC7300067; doi:10.1002/fsn3.1542)

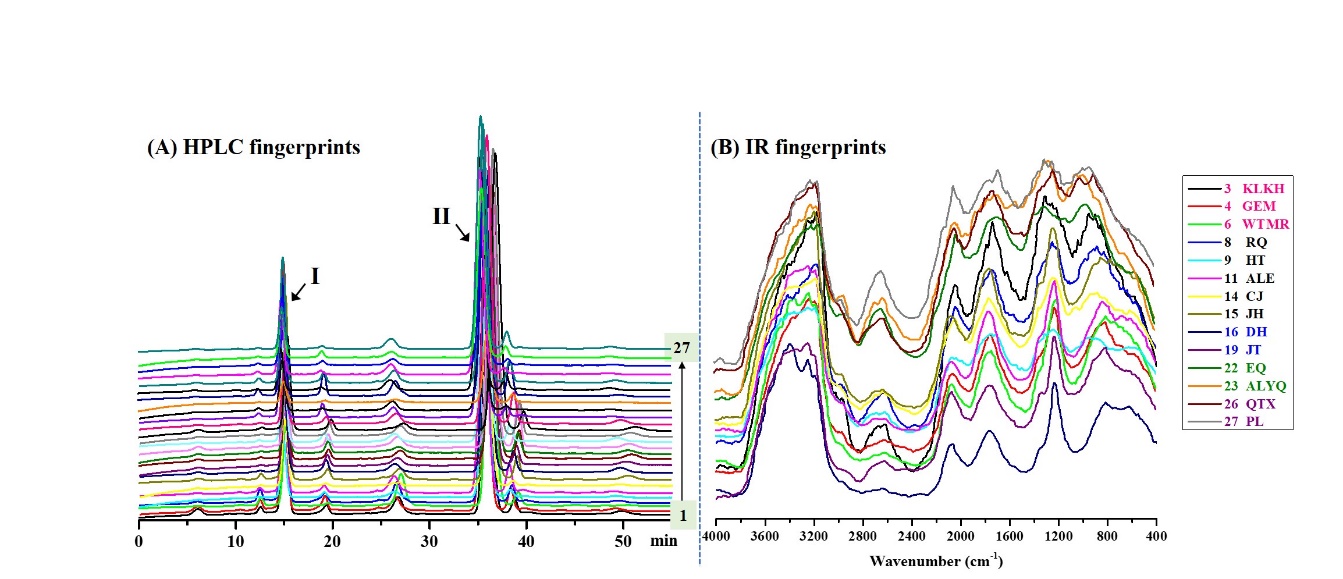


**Figure S5** The HPLC and IR fingerprints of LRAE.

Supplement: Supplementary file 5 — Figure S5 [file FSN3-8-2642-s005.docx]
