# Supplementary material for: Ultrasonic extraction of anthocyanins from Lycium ruthenicum Murr. and its antioxidant activity
Source: Food Sci Nutr. 2020 Apr 27;8(6):2642–51. doi: 10.1002/fsn3.1542 (PMC7300067; doi:10.1002/fsn3.1542)
Supplement: Supplementary file 6 — Figure S6 [file FSN3-8-2642-s006.docx]

**Figure S6** Scavenging activity of LRAE obtained from five regions (KLKH, CJ, DH, EQ and PL). The concentration of LRAE was 1.0 mg/mL. W: wild LR fruits, C: cultivated LR fruits.
