# Supplementary material for: Ultrasonic extraction of anthocyanins from Lycium ruthenicum Murr. and its antioxidant activity
Source: Food Sci Nutr. 2020 Apr 27;8(6):2642–51. doi: 10.1002/fsn3.1542 (PMC7300067; doi:10.1002/fsn3.1542)
Supplement: Supplementary file 7 — Table S1 [file FSN3-8-2642-s007.docx]

**Table S1** Response values for the anthocyanin (PRG) content via ultrasonic extraction based on BBD.

| Run | Independent variable | | | |  | PRG content (mg/g DW) | |
| --- | --- | --- | --- | --- | --- | --- | --- |
|  | *X_1_* (power, W) | *X_2_* (extractant - material ratio, mL/g) | *X_3_* (temperature,  ℃) | *X_4_* (time, min) |  | Experimental | Predicted |
| 1 | -1(100) | -1(10) | 0(40) | 0(25) |  | 25.32 | 24.68 |
| 2 | +1(500) | -1 | 0 | 0 |  | 25.63 | 25.57 |
| 3 | -1 | +1(30) | 0 | 0 |  | 26.78 | 26.28 |
| 4 | +1 | +1 | 0 | 0 |  | 27.54 | 27.62 |
| 5 | 0(300) | 0(20) | -1(30) | -1(15) |  | 24.46 | 24.12 |
| 6 | 0 | 0 | +1(50) | -1 |  | 25.75 | 25.48 |
| 7 | 0 | 0 | -1 | +1(35) |  | 27.04 | 26.75 |
| 8 | 0 | 0 | +1 | +1 |  | 27.73 | 27.51 |
| 9 | -1 | 0 | 0 | -1 |  | 23.40 | 23.64 |
| 10 | +1 | 0 | 0 | -1 |  | 25.26 | 25.43 |
| 11 | -1 | 0 | 0 | +1 |  | 26.41 | 26.64 |
| 12 | +1 | 0 | 0 | +1 |  | 26.93 | 27.09 |
| 13 | 0 | -1 | -1 | 0 |  | 24.72 | 24.50 |
| 14 | 0 | +1 | -1 | 0 |  | 26.74 | 27.05 |
| 15 | 0 | -1 | +1 | 0 |  | 26.20 | 26.29 |
| 16 | 0 | +1 | +1 | 0 |  | 26.77 | 27.39 |
| 17 | -1 | 0 | -1 | 0 |  | 24.36 | 24.88 |
| 18 | +1 | 0 | -1 | 0 |  | 26.57 | 26.58 |
| 19 | -1 | 0 | +1 | 0 |  | 26.39 | 26.53 |
| 20 | +1 | 0 | +1 | 0 |  | 27.42 | 27.06 |
| 21 | 0 | -1 | 0 | -1 |  | 23.07 | 23.50 |
| 22 | 0 | +1 | 0 | -1 |  | 25.90 | 25.66 |
| 23 | 0 | -1 | 0 | +1 |  | 25.76 | 26.16 |
| 24 | 0 | +1 | 0 | +1 |  | 27.93 | 27.66 |
| 25 | 0 | 0 | 0 | 0 |  | 28.12 | 27.74 |
| 26 | 0 | 0 | 0 | 0 |  | 27.94 | 27.74 |
| 27 | 0 | 0 | 0 | 0 |  | 27.65 | 27.74 |
| 28 | 0 | 0 | 0 | 0 |  | 27.11 | 27.74 |
| 29 | 0 | 0 | 0 | 0 |  | 27.90 | 27.74 |
