# Supplementary material for: Ultrasonic extraction of anthocyanins from Lycium ruthenicum Murr. and its antioxidant activity
Source: Food Sci Nutr. 2020 Apr 27;8(6):2642–51. doi: 10.1002/fsn3.1542 (PMC7300067; doi:10.1002/fsn3.1542)
Supplement: Supplementary file 8 — Table S2 [file FSN3-8-2642-s008.docx]

**Table S2** Contents of anthocyanidins (delphinidin, petunidin and malvidin) in wild and cultivated LR fruits from different regions in China.

| No. | Locality | Code | Delphinidin (mg/g) | | Petunidin (mg/g) | | Malvidin (mg/g) | |
| --- | --- | --- | --- | --- | --- | --- | --- | --- |
|  |  |  | Wild | Cultivated | Wild | Cultivated | Wild | Cultivated |
| 1 | Xiangride | XRD | 0.20 ± 0.01 | 0.23 ± 0.00 | 6.55 ± 0.31 | 6.64 ± 0.27 | 0.49 ± 0.03 | 0.55 ± 0.02 |
| 2 | Nuomuhong | NMH | 0.42 ± 0.05 | 0.41 ± 0.03 | 8.43 ± 0.29 | 7.56 ± 0.33 | 0.30 ± 0.01 | 0.57 ± 0.01 |
| 3 | KeluKehu | KLKH | 1.12 ± 0.11 | 0.49 ± 0.01 | 9.36 ± 0.54 | 7.85 ± 0.25 | 0.32 ± 0.00 | 0.54 ± 0.01 |
| 4 | Geermu | GEM | 0.50 ± 0.02 | 1.03 ± 0.04 | 10.78 ± 0.37 | 13.81 ± 1.15 | 0.77 ± 0.03 | 0.84 ± 0.09 |
| 5 | Dagele | DGL | 0.30 ± 0.00 | 0.17 ± 0.00 | 5.64 ± 0.21 | 3.56 ± 0.11 | 0.35 ± 0.01 | 0.33 ± 0.01 |
| 6 | Urt Moron | WTMR | 0.46 ± 0.08 | 0.52 ± 0.07 | 8.71 ± 0.44 | 8.98 ± 0.28 | 0.45 ± 0.02 | 0.70 ± 0.05 |
| 7 | Hongliugou | HLG | 0.07 ± 0.00 | 0.14 ± 0.00 | 1.74 ± 0.22 | 4.27 ± 0.10 | 0.28 ± 0.00 | 0.52 ± 0.06 |
| 8 | Ruoqiang | RQ | 0.15 ± 0.00 | 0.47 ± 0.05 | 5.44 ± 0.17 | 10.74 ± 0.56 | 0.65 ± 0.15 | 1.02 ± 0.12 |
| 9 | Hetian | HT | 0.09 ± 0.00 | 0.16 ± 0.00 | 3.97 ± 0.21 | 4.57 ± 0.32 | 0.48 ± 0.08 | 0.52 ± 0.03 |
| 10 | Kashgar | KS | 0.07 ± 0.00 | 0.14 ± 0.00 | 4.06 ± 0.18 | 4.78 ± 0.14 | 0.30 ± 0.02 | 0.54 ± 0.02 |
| 11 | Alaer | ALE | 0.19 ± 0.01 | 0.29 ± 0.02 | 5.31 ± 0.12 | 2.98 ± 0.06 | 0.35 ± 0.01 | 0.33 ± 0.01 |
| 12 | Yuli | YL | 0.07 ± 0.00 | 0.07 ± 0.00 | 2.34 ± 0.09 | 4.32 ± 0.11 | 0.28 ± 0.01 | 0.51 ± 0.05 |
| 13 | Turpan | TUP | 0.24 ± 0.02 | 0.34 ± 0.03 | 7.05 ± 0.19 | 8.42 ± 0.20 | 0.55 ± 0.03 | 0.83 ± 0.07 |
| 14 | Changji | CJ | 0.10 ± 0.00 | 0.11 ± 0.00 | 4.50 ± 0.22 | 4.89 ± 0.34 | 0.35 ± 0.02 | 0.38 ± 0.04 |
| 15 | Jinghe | JH | 0.22 ± 0.01 | 0.20 ± 0.00 | 5.94 ± 0.27 | 8.09 ± 0.65 | 0.76 ± 0.02 | 0.80 ± 0.10 |
| 16 | Dunhuang | DH | 0.20 ± 0.00 | 0.16 ± 0.00 | 4.11 ± 0.13 | 5.07 ± 0.17 | 0.38 ± 0.01 | 0.40 ± 0.05 |
| 17 | Guazhou | GZ | 0.18 ± 0.00 | 0.23 ± 0.01 | 5.02 ± 0.24 | 7.35 ± 0.34 | 0.60 ± 0.03 | 0.75 ± 0.04 |
| 18 | Jiayuguan | JYG | 0.15 ± 0.00 | 0.12 ± 0.00 | 5.79 ± 0.33 | 5.65 ± 0.08 | 0.40 ± 0.01 | 0.42 ± 0.01 |
| 19 | Jinta | JT | 0.07 ± 0.00 | 0.08 ± 0.00 | 2.36 ± 0.09 | 2.91 ± 0.06 | 0.33 ± 0.02 | 0.31 ± 0.03 |
| 20 | Shandan | SD | 0.10 ± 0.00 | 0.08 ± 0.00 | 3.50 ± 0.26 | 3.24 ± 0.17 | 0.31 ± 0.00 | 0.35 ± 0.01 |
| 21 | Minqin | MQ | 0.34 ± 0.03 | 0.38 ± 0.04 | 5.88 ± 0.24 | 5.32 ± 0.12 | 0.45 ± 0.03 | 0.44 ± 0.02 |
| 22 | EjinaQi | EQ | 0.24 ± 0.01 | 0.20 ± 0.00 | 4.33 ± 0.30 | 4.64 ± 0.12 | 0.28 ± 0.01 | 0.37 ± 0.04 |
| 23 | AlxaYouqi | ALYQ | 0.44 ± 0.03 | 0.50 ± 0.02 | 6.37 ± 0.52 | 5.85 ± 0.28 | 0.39 ± 0.02 | 0.34 ± 0.03 |
| 24 | Alxa Zuoqi | ALZQ | 0.40 ± 0.01 | 0.42 ± 0.00 | 5.30 ± 0.38 | 5.44 ± 0.32 | 0.35 ± 0.01 | 0.31 ± 0.01 |
| 25 | Bayan Nur | BYN | 0.25 ± 0.00 | 0.39 ± 0.01 | 4.82 ± 0.32 | 5.16 ± 0.23 | 0.30 ± 0.00 | 0.37 ± 0.03 |
| 26 | Qingtongxia | QTX | 0.37 ± 0.04 | 0.39 ± 0.02 | 4.60 ± 0.19 | 4.97 ± 0.22 | 0.32 ± 0.01 | 0.35 ± 0.01 |
| 27 | Pingluo | PL | 0.25 ± 0.01 | 0.40 ± 0.02 | 4.48 ± 0.17 | 4.71 ± 0.15 | 0.43 ± 0.02 | 0.45 ± 0.05 |
